# Cumulative burden of maternal vascular malperfusion and its association with early cerebral oxygenation in neonates

**DOI:** 10.3389/fcell.2026.1756278

**Published:** 2026-02-11

**Authors:** Yao Zhang, Tong Yang, Jiaxi Wu, Yanxia Mao, Jingxian Shi, Haoran Dou, Jinhui Li, Jun Tang, Tao Xiong

**Affiliations:** 1 Department of Pediatrics, West China Second University Hospital, Sichuan University, Chengdu, China; 2 Key Laboratory of Birth Defects and Related Diseases of Women and Children (Sichuan University), Ministry of Education, Chengdu, China; 3 Children’s Medicine Key Laboratory of Sichuan Province, Chengdu, China

**Keywords:** cerebral oxygenation, maternal vascular malperfusion, near-infrared spectroscopy, neonates, placental pathology

## Abstract

**Objectives:**

Maternal vascular malperfusion (MVM) represents a major cause of chronic fetal hypoxia and is associated with adverse neonatal outcomes. However, whether the cumulative burden of MVM lesions influences early cerebral oxygenation in neonates remains unclear. This study aimed to evaluate the association between the number of placental MVM lesion types and neonatal cerebral regional oxygen saturation (crSO_2_) and cerebral fractional tissue oxygen extraction (cFTOE).

**Methods:**

In this retrospective observational study, 508 neonates admitted between January 2021 and March 2024 were included. Based on placental histopathology, infants were categorized into three groups according to the number of MVM lesion types: no MVM, 1–2 MVM types, and 3–5 MVM types. Cerebral oxygenation was monitored weekly from birth to postnatal day 28 using near-infrared spectroscopy. Multivariable linear regression models were used to assess associations between MVM burden and crSO_2_/cFTOE during the first postnatal week, adjusting for relevant maternal and neonatal covariates.

**Results:**

Compared with neonates without MVM, those with placental findings of 3–5 types of MVM had significantly higher crSO_2_ (β = 1.65; 95% CI, 0.05–3.24; P = 0.044) and lower cFTOE (β = −0.02; 95% CI, −0.04 to −0.01; P = 0.029) during the first postnatal week. No significant differences were observed in the 1–2 MVM group. Longitudinal analyses demonstrated decreasing crSO_2_ and increasing cFTOE with advancing postnatal age across all groups, with the early differences between groups attenuating over time.

**Conclusion:**

A cumulative burden of 3–5 MVM types is independently associated with altered cerebral oxygenation patterns in the early neonatal period, characterized by higher crSO_2_ and lower cFTOE. These findings suggest that the cumulative burden of maternal vascular malperfusion lesions identified on postpartum placental examination may provide clinically relevant contextual information for interpreting early neonatal cerebral oxygenation patterns and underscore the need for enhanced physiological monitoring during the first postnatal week.

## 1 Introduction

The placenta plays a central role in regulating the exchange of oxygen and nutrients between the maternal and fetal circulations and is therefore critical for fetal growth, development, and neonatal outcomes (Carbillon et al., 2022; Burton et al., 2016; Zur et al., 2024; Kulkarni et al., 2021). When placental function is impaired, the fetus may be exposed to chronic hypoxia, which contributes to stillbirth, fetal growth restriction (FGR), and adverse neonatal conditions (Silver, 2018). A key pathological mechanism underlying placental dysfunction is maternal vascular malperfusion (MVM), characterized by impaired maternal blood flow to the intervillous space. MVM reflects defective spiral artery remodeling and abnormal placental vascular development, and is a hallmark lesion in pregnancies complicated by hypertensive disorders, FGR, and placental insufficiency (Zur et al., 2020).

Chronic intrauterine hypoxia has profound consequences for fetal brain development. To preserve oxygen delivery to vital organs, the fetus initiates a compensatory “brain-sparing effect,” redistributing blood flow toward the cerebral circulation (Miller et al., 2016). Although initially protective, prolonged hypoxia may exceed compensatory capacity and lead to structural and functional alterations of the developing brain, including reduced brain volume and white matter injury (Su et al., 2015; Nijman et al., 2024; Alves de Alencar Rocha et al., 2017). These disturbances are closely associated with long-term neurodevelopmental disorders in offspring, such as cognitive deficits and motor dysfunction (Sadhwani et al., 2022; Spinillo et al., 2021; Ibrahim et al., 2025). Prior studies have demonstrated that the degree of placental pathology correlates with the risk of neonatal brain injury and later neurodevelopmental abnormalities, suggesting that placental lesions may serve as early markers of neurological vulnerability (Spinillo et al., 2023).

Notably, the severity of placental injury may exhibit a cumulative effect—specifically, a greater number of pathological subtypes of MVM correlates with a more pronounced impact on adverse neonatal outcomes.

Near-infrared spectroscopy (NIRS) permits continuous, non-invasive assessment of cerebral regional oxygen saturation (crSO_2_) and cerebral fractional tissue oxygen extraction (cFTOE), thereby reflecting the balance between cerebral oxygen supply and consumption. These parameters have been associated with the prediction of neonatal brain injury and long-term outcomes (Chock et al., 2020; Ćaleta et al., 2024). However, few studies have examined how placental pathology influences postnatal cerebral oxygenation. Moreover, to our knowledge, no study has evaluated whether the cumulative burden of MVM lesions—reflected by the number of distinct pathological subtypes—affects cerebral oxygenation patterns in neonates.

Prior study indicates that multiple placental lesions can exert additive or synergistic effects on pregnancy outcomes, and evaluating the cumulative burden of MVM may provide a more accurate representation of placental dysfunction than assessing individual lesions alone (Arts et al., 2022). Whether such cumulative pathological load translates into measurable differences in early cerebral oxygen delivery and utilization in neonates remains unknown.

Therefore, the present study aimed to investigate the association between the cumulative burden of placental MVM and early cerebral oxygenation in neonates. By comparing longitudinal crSO_2_ and cFTOE patterns across infants with varying degrees of MVM burden, we sought to determine whether increasing placental vascular pathology is associated with altered cerebral oxygen metabolism during the critical early postnatal period. Such findings may help clarify mechanisms linking placental dysfunction to neonatal brain vulnerability and support the use of placental pathology as an early indicator for targeted cerebral monitoring and neurodevelopmental risk stratification.

## 2 Methods

### 2.1 Study design and population

This retrospective observational study included 508 neonates admitted to the Department of Neonatology at West China Second University Hospital between 1 January 2021 and 1 March 2024. Eligible neonates were those who underwent crSO_2_ monitoring during hospitalization and whose mothers received placental histopathological examination.

#### 2.1.1 Inclusion criteria

Neonates were included if they met the following conditions: (1) Complete maternal clinical data and placental pathological reports available; (2) Singleton pregnancy; (3) Availability of crSO_2_ monitoring data after admission to the neonatal ward.

#### 2.1.2 Exclusion criteria

Neonates were excluded for any of the following: (1) Multiple gestations; (2) Known genetic or metabolic diseases; (3) Major congenital anomalies.

**FIGURE 1:**
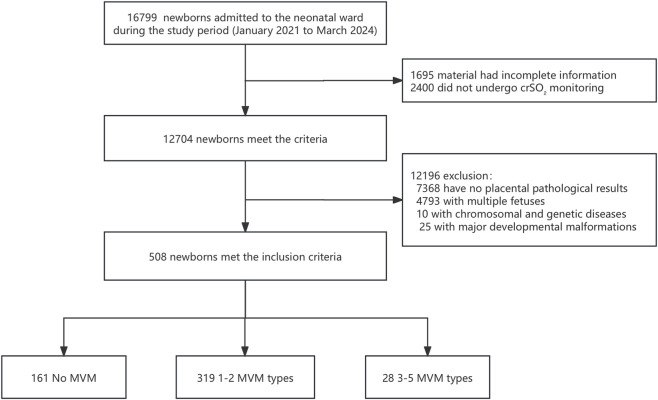
Screening flow chart. This study included 508 neonates admitted to the Department of Neonatology at West China Second University Hospital between 1 January 2021 and 1 March 2024. Eligible neonates were those who underwent crSO_2_ monitoring during hospitalization and whose mothers received placental histopathological examination. Based on the number of pathological subtypes identified, infants were classified into: No MVM group, 1–2 MVM subtypes group, and 3–5 MVM subtypes group.

A total of 508 neonates met the eligibility criteria. Among them, 161 had no MVM, and 347 had one or more MVM lesion types. Based on the number of pathological subtypes identified, infants were classified into: No MVM group, 1–2 MVM subtypes group, and 3–5 MVM subtypes group (Figure 1).

The study was approved by the Ethics Committee of West China Second University Hospital (Approval No. Z-2019-41-2101-04) and adhered to the STROBE (Strengthening the Reporting of Observational Studies in Epidemiology) guidelines.

### 2.2 Placental pathological examination

Following placental delivery, the specimen was immediately transported to the Department of Pathology by designated personnel. A board-certified pathologist performed macroscopic evaluations, including weighing the placenta, measuring its volume and umbilical cord length, inspecting the surface for gross abnormalities, and documenting findings with standardized photography. The maternal surface of the placenta was incised at 1–2 cm intervals using sterile scissors, and the tissue was fixed in 10% neutral-buffered formalin for 24 h. After fixation, the specimen was re-examined for evidence of infarction, hematoma, or other pathological lesions. For microscopic analysis, at least four tissue samples were collected: two from the amniotic membrane, two from macroscopically normal placental parenchyma, and additional samples from any grossly visible lesions.

Diagnostic assessments were conducted by attending pathologists at the Department of Pathology, West China Second University Hospital, Sichuan University, with all findings reviewed and confirmed by a senior pathologist. MVM was categorized into six subtypes: decidual vasculopathy, placental infarcts, distal villous hypoplasia, accelerated villous maturation, retroplacental hemorrhage, and placental hypoplasia (Khong et al., 2016). Since placental hypoplasia was not observed in the study cohort, it was excluded from subsequent analyses. All placental pathological examination procedures and diagnostic criteria adhered to the 2016 Consensus of the Amsterdam Placenta Workshop Group.

Study participants were stratified into three groups based on the number of MVM subtypes present: the no MVM, the 1–2 MVM subtypes group, and the 3–5 MVM subtypes group (Arts et al., 2022).

Data on selected fetal vascular malperfusion and inflammatory placental lesions were extracted when available and are summarized in Supplementary Table S1.

#### 2.2.1 Data collection

##### 2.2.1.1 Neonatal data collection

The crSO_2_ was assessmented on all infants admitted to the Department of Neonatology, West China Second University Hospital, between 1 January 2021, and 1 March 2024. Demographic and clinical data were collected for all enrolled neonates, including: Baseline characteristics: Sex, ethnicity, parity, gravidity, gestational age, birth weight, body length, and Apgar scores; Therapeutic interventions: Use of mechanical ventilation, blood transfusion, and pharmacotherapies (e.g., caffeine); Neonatal morbidities: Apnea, gastroin testinal bleeding, necrotizing enterocolitis, small for gestational age, neonatal respiratory distress syndrome, bronchopulmonary dysplasia, retinopathy of prematurity, sepsis, intracranial hemorrhage, and patent ductus arteriosus.

##### 2.2.1.2 Maternal data collection

Per the clinical pathway of the Obstetrics Department of our hospital, histopathological examination of the placenta is not a routine procedure. Placental submission for pathological assessment is typically warranted based on the following indications: maternal comorbidities of severe pregnancy-related complications, including gestational hypertension and gestational diabetes mellitus; abnormal intrapartum events such as fetal heart rate abnormalities, severe meconium-stained amniotic fluid, placental abruption, and placenta previa; neonatal conditions including preterm birth and small for gestational age (SGA); grossly visible placental abnormalities identified during or post-delivery, such as infarctions, thrombi, or morphological anomalies. Demographic and clinical data were collected for all enrolled maternal, including: Baseline characteristics: Maternal age, pre-pregnancy body mass index, gestational weight gain, and pre-delivery blood pressure; Placental pathological findings: Decidual vasculopathy, placental infarcts, distal villous hypoplasia, accelerated villous maturation, retroplacental hemorrhage, and placental hypoplasia (excluded from analysis as no cases were observed); Pregnancy complications: Fetal distress, fetal growth restriction, maternal obesity, advanced maternal age at first pregnancy, hypoproteinemia, gestational hypothyroidism, gestational hyperthyroidism, gestational diabetes mellitus, hypertensive disorders of pregnancy (HDP), and intrahepatic cholestasis of pregnancy.

#### 2.2.2 Outcome indicators

##### 2.2.2.1 crSO_2_

The EGOS-600B NIRS device (ENGINMED Co., China) was used to estimated crSO_2_. NIRS estimated regional tissue oxygen saturation by measuring the oxygenated hemoglobin (HbO_2_) and deoxygenated hemoglobin (Hb) in local tissues. The calculated value is expressed as: crSO_2_ = [HbO_2_/(HbO_2_ + Hb)] × 100%. Trained personnel should adhere to standard operating procedures by securing the NIRS probe at the mid-forehead using an elastic bandage while the neonatal remains in a quiet, supine state (He et al., 2024). Measurements commence once the signal has stabilized. Each measurement session lasts 20 min, with data recorded at 5-min intervals. The final value for analysis is derived from the average of the five recorded readings. This assessment is performed weekly throughout hospitalization until either discharge or the 28th postnatal day, whichever occurred first.

##### 2.2.2.2 cFTOE

cFTOE reflects the balance between oxygen supply to the brain and oxygen consumption.

cFTOE= (peripheral arterial oxygen saturation - crSO_2_)/(peripheral arterial oxygen saturation).

### 2.3 Statistical methods

Data analysis was conducted using SPSS statistical software (version 27.1). Continuous variables that met the assumption of normality were summarized as mean ± standard deviation (SD), whereas non-normally distributed data were presented as median and interquartile range (IQR). To initially explore intergroup differences, we first performed unadjusted comparisons of continuous variables (e.g., crSO_2_ and cFTOE) across the three MVM groups. For normally distributed data, one-way analysis of variance (ANOVA) was employed; for non-normally distributed data, the Kruskal-Wallis H test was utilized. To evaluate the independent association between MVM burden and cerebral oxygenation parameters, while controlling for potential confounding factors, we further constructed multivariate linear regression models. In the regression analyses, MVM severity (stratified by the number of pathological subtypes) was included as the primary independent variable, with crSO_2_ and cFTOE (measured within the first week of life) serving as dependent variables.

Covariates included in the multivariate linear regression models were selected based on *a priori* clinical knowledge and existing literature evidence. The specific rationale for inclusion is detailed as follows:

Maternal demographic factors: Maternal age and pre-pregnancy body mass index (BMI) were included as baseline factors, given their established associations with maternal vascular health and pregnancy outcomes.

Pregnancy complications: Gestational hypertensive disorders (including preeclampsia), a core clinical phenotype of MVM, were included as they directly correlate with impaired placental perfusion and thus represent a critical confounding variable. Gestational diabetes mellitus (GDM) and intrahepatic cholestasis of pregnancy (ICP)—common gestational metabolic disorders—were included, as they may indirectly influence fetal/neonatal oxygenation status by modulating maternal systemic inflammation, oxidative stress, or placental function.

Endocrine disorders: Hypothyroidism (both clinical and subclinical) was included, given its links to adverse pregnancy outcomes and elevated neurodevelopmental risk in offspring, which may constitute a potential confounding pathway.

The inclusion of these covariates was intended to adjust for factors known or suspected to be associated with both the presence of MVM burden and the outcome (neonatal cerebral oxygenation), thereby enabling a more precise estimation of the independent effect of MVM burden on cerebral oxygenation.

## 3 Results

A total of 508 neonates were included in the final analysis. Based on the number of MVM lesion subtypes, 161 infants (31.7%) were classified into the no MVM group, 319 (62.8%) into the 1–2 MVM subtypes group, and 28 (5.5%) into the 3–5 MVM subtypes group.

### 3.1 Neonatal characteristics

Neonatal baseline characteristics are presented in Table 1. There were no significant differences among groups in gestational age, birth weight, sex, small-for-gestational-age status, gastrointestinal bleeding, apnea, sepsis, necrotizing enterocolitis, intraventricular hemorrhage, patent ductus arteriosus, retinopathy of prematurity, or bronchopulmonary dysplasia (all P > 0.05).

**TABLE 1:** Neonatal characteristics across MVM burden groups.

Variables	No MVM (n = 161)	1–2 MVM types (n = 319)	3-5 MVM types (n = 28)	*P*
Gestational age, weeks, M (Q_1_, Q_3_)	34.00 (31.86,35.00)	34.00 (31.93,35.50)	33.36 (32.25,34.93)	0.586
Birth weight, g, M (Q_1_, Q_3_)	1840.00 (1,490.00,2300.00)	1880.00 (1,410.00,2295.00)	1,695.00 (1,370.00,2145.00)	0.456
Male, n (%)	81 (50.31)	166 (52.04)	13 (46.43)	0.821
Gastrointestinal bleeding, n (%)	6 (3.73)	10 (3.13)	1 (3.57)	0.942
Apnea, n (%)	5 (3.11)	12 (3.76)	0 (0.00)	0.558
Sepsis, n (%)	24 (14.91)	31 (9.72)	1 (3.57)	0.100
Necrotizing enterocolitis, n (%)	2 (1.24)	7 (2.19)	0 (0.00)	0.835
Small for gestational age, n (%)	30 (18.63)	72 (22.57)	10 (35.71)	0.123
Respiratory distress syndrome, n (%)	77 (47.83)	108 (33.86)	11 (39.29)	0.012
Bronchopulmonary dysplasia, n (%)	13 (8.07)	20 (6.27)	2 (7.14)	0.761
Retinopathy of prematurity, n (%)	9 (5.59)	26 (8.15)	1 (3.57)	0.445
Intraventricular haemorrhage, n (%)	29 (18.12)	44 (13.79)	6 (21.43)	0.318
Patent ductus arteriosus, n (%)	65 (40.37)	136 (42.63)	14 (50.00)	0.625
Fetal distress, n (%)	2 (1.24)	2 (0.63)	0 (0.00)	0.685

MVM, maternal vascular malperfusion.

The incidence of respiratory distress syndrome differed significantly across groups (P = 0.012).

### 3.2 Maternal and placental characteristics

Maternal clinical characteristics are shown in Table 2. Maternal age, Body Mass Index (BMI) before delivery, hypothyroidism, and intrahepatic cholestasis of pregnancy did not differ significantly among groups (all P > 0.05).

**TABLE 2:** Maternal characteristics across MVM burden groups.

Variables	No MVM (n = 161)	1–2 MVM types (n = 319)	3–5 MVM types (n = 28)	*P*
Maternal age, M (Q_1_, Q_3_)	33.00 (30.00,36.00)	31.00 (29.00,35.00)	32.50 (28.75,34.25)	0.064
BMI before delivery, kg/m^2^, M (Q_1_, Q_3_)	25.56 (23.53,28.55)	25.90 (23.62,28.21)	25.04 (23.64,26.75)	0.581
Fetal distress, n (%)	2 (1.24)	2 (0.63)	0 (0.00)	0.685
Hypothyroidism, n (%)	27 (16.77)	49 (15.36)	4 (14.29)	0.901
GDM, n (%)	70 (43.48)	97 (30.41)	11 (39.29)	0.016
Intrahepatic cholestasis of pregnancy, n (%)	14 (8.70)	21 (6.58)	3 (10.71)	0.566
HDP, n (%)	65 (40.37)	141 (44.20)	20 (71.43)	0.009

MVM, maternal vascular malperfusion; BMI, body mass index; GDM, gestational diabetes mellitus; HDP, hypertensive disorders of pregnancy.

Two pregnancy complications showed significant differences: Gestational diabetes mellitus (GDM): 43.48% (no MVM), 30.41% (1–2 MVM), 39.29% (3–5 MVM); P = 0.016; Hypertensive disorders of pregnancy (HDP): 40.37% (no MVM), 44.20% (1–2 MVM), 71.43% (3–5 MVM); P = 0.009.

**FIGURE 2:**
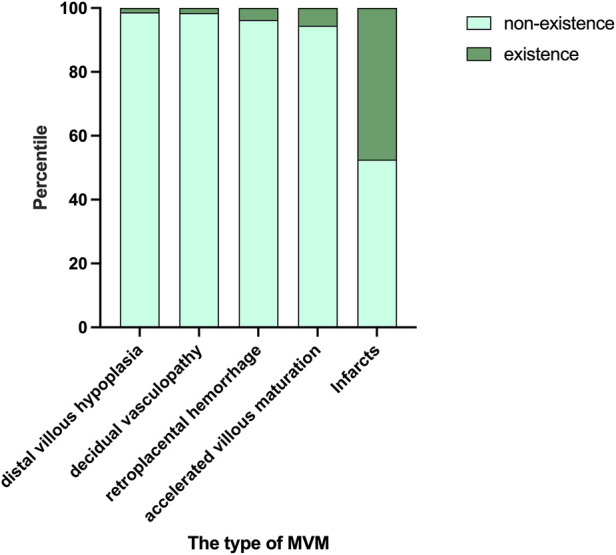
The occurrence of various pathological types. Placental infarction was the most common lesion (47.44%), followed by accelerated villous maturation (5.51%), retroplacental hemorrhage (3.74%), decidual vasculopathy (1.57%), distal villous hypoplasia (1.38%).

Placental measurements (weight, length, width, thickness, volume) did not differ among groups (Table 3). Placental infarction was the most common lesion (47.44%), followed by accelerated villous maturation (5.51%), retroplacental hemorrhage (3.74%), decidual vasculopathy (1.57%) and distal villous hypoplasia (1.38%) (Figure 2).

**TABLE 3:** Placental measurements across MVM burden groups.

Variables	No MVM (n = 161)	1–2 MVM types (n = 319)	3–5 MVM types (n = 28)	*P*
Weight (g), M (Q_1_, Q_3_)	343.00 (276.50,413.50)	360.00 (289.00,445.00)	345.50 (268.00,436.00)	0.461
Length (cm), M (Q_1_, Q_3_)	16.00 (14.35,17.05)	16.00 (15.00,17.57)	15.00 (14.00,16.50)	0.260
Width (cm), M (Q_1_, Q_3_)	13.00 (11.90,15.00)	13.50 (12.00,15.00)	12.50 (11.27,13.88)	0.430
Thickness (cm), M (Q_1_, Q_3_)	2.70 (2.23,3.00)	2.50 (2.00,3.00)	2.55 (2.05,3.00)	0.362
Volume (cm^3^), M (Q_1_, Q_3_)	550.20 (442.53,718.76)	566.25 (416.00,720.00)	529.08 (378.75,626.14)	0.429

MVM, maternal vascular malperfusion.

### 3.3 Longitudinal changes in cerebral oxygenation

#### 3.3.1 crSO_2_

**FIGURE 3:**
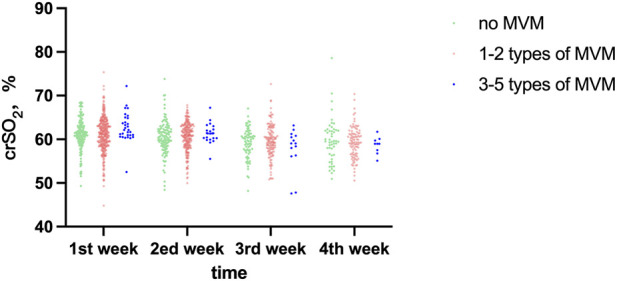
Comparative analysis of crSO_2_ across different time points among study groups.

crSO_2_ declined progressively with postnatal age (time effect: F = 7.242, P < 0.001). There were no significant differences between the MVM groups at any of the four measured time points (all P > 0.05). The time × group interaction did not reach statistical significance (F = 1.918, P = 0.080). The distribution of crSO_2_ in each group at different time points is presented in Table 4 and Figure 3.

**TABLE 4:** crSO_2_ values across postnatal weeks.

Variables	No MVM (n = 161)	1-2 MVM types (n = 319)	3-5 MVM types (n = 28)	*P*
1st week, %, M (Q_1_, Q_3_)	61.23 (59.56,63.01)	61.23 (58.77,63.56)	62.16 (60.85,64.89)	0.059
2 nd week, %,M (Q_1_, Q_3_)	60.46 (58.53,62.34)	60.92 (58.62,63.00)	61.35 (60.20,62.45)	0.367
3rd week, %,M (Q_1_, Q_3_)	59.73 (57.41,61.29)	59.46 (57.62,61.83)	59.05 (56.27,60.90)	0.690
4th week, %,M (Q_1_, Q_3_)	59.44 (56.37,61.97)	59.32 (56.65,61.29)	58.96 (57.16,59.91)	0.845
Between-group effects			F = 0.078, P = 0.925	
Within-group effect (time effect)			F = 7.242, P < 0.001	
Interaction effects			F = 1.918, P = 0.080	

MVM, maternal vascular malperfusion; crSO_2_, cerebral regional oxygen saturation.

#### 3.3.2 cFTOE

**FIGURE 4:**
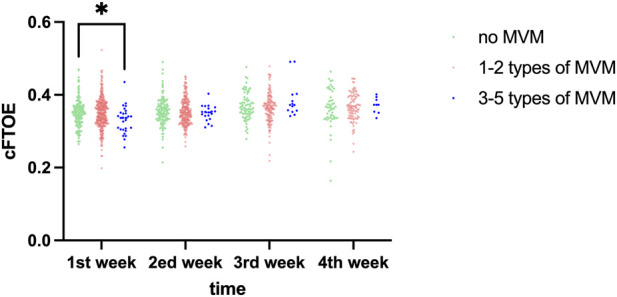
Comparative analysis of cFTOE across different time points among study groups. *,p < 0.05.

cFTOE increased consistently across postnatal weeks (time effect: F = 6.712, P < 0.001). During the first postnatal week, notable disparities in cFTOE were observed among the groups (P = 0.026), post-hoc pairwise comparisons that the cFTOE in the 3–5 MVM group was significantly lower than both the No MVM group (P = 0.044) and the 1–2 MVM group (P = 0.021), while no difference was observed between the No MVM and 1–2 MVM groups (P > 0.05). No significant differences were found from week 2 to week 4 (all P > 0.05). The time × group interaction was not significant (F = 1.716, P = 0.183). The distribution of cFTOE in each group at different time points is presented in Table 5 and Figure 4.

**TABLE 5:** cFTOE values across postnatal weeks.

Variables	No MVM (n = 161)	1-2MVM types (n = 319)	3–5 MVM types (n = 28)	*P*
1st week, M (Q_1_, Q_3_)	0.3505 (0.3253,0.3681)	0.3499 (0.3238,0.3760)	0.3368 (0.3052,0.3499)	0.026^a^
2nd week, M (Q_1_, Q_3_)	0.3541 (0.3338,0.3745)	0.3509 (0.3294,0.3772)	0.3525 (0.3401,0.3635)	0.750
3rd week, M (Q_1_, Q_3_)	0.3625 (0.3477,0.3859)	0.3648 (0.3408,0.3843)	0.3722 (0.3559,0.4014)	0.468
4th week, M (Q_1_, Q_3_)	0.3649 (0.3370,0.3893)	0.3657 (0.3412,0.3960)	0.3728 (0.3523,0.3903)	0.906
Between-group effects		F = 0.261, P = 0.771		
Within-group effect (time effect)		F = 6.712, P < 0.001		
Interaction effects		F = 1.716, P = 0.183		

MVM, maternal vascular malperfusion; cFTOE, cerebral fractional tissue oxygen extraction.

^a^ Post-hoc pairwise comparisons for cFTOE, at week 1: 3-5 MVM, types vs. No MVM:P = 0.044; 3-5 MVM, types vs.1-2 MVM, types: P = 0.021; No MVM, vs. 1-2 MVM, types: P > 0.05.

### 3.4 Association between MVM burden and cerebral oxygenation

#### 3.4.1 crSO_2_ during the first postnatal week

Univariate linear regression analysis showed that compared with no MVM, 3-5 types of MVM group were associated with higher crSO_2_ within the first week after birth (β: 1.686, 95%CI: 0.151–3.220, P = 0.032), while 1-2 types of MVM were not significant. After adjusting for confounding factors such as gestational diabetes mellitus, gestational hypertension, and hypothyroidism, the presence of placental findings of 3-5 types of MVM was associated with higher crSO_2_ within the first week of life compared with no MVM (β: 1.648, 95%CI: 0.051–3.245, P = 0.044) (Table 6).

**TABLE 6:** Linear regression analysis of crSO_2_ and cFTOE during the first postnatal week.

Variables	Unadjusted				Adjusted^a^			
	S.E	t	*P*	β (95%CI)	S.E	t	*P*	β (95%CI)
crSO_2_								
No MVM	Ref				Ref			
1–2 MVM types	0.373	−0.179	0.858	−0.067 (−0.798, 0.664)	0.385	−0.342	0.732	−0.132 (−0.886, 0.622)
3-5MVM types	0.783	2.153	0.032	1.686 (0.151, 3.220)	0.815	2.022	0.044	1.648 (0.051, 3.245)
cFTOE								
No MVM	Ref				Ref			
1–2 MVM types	0.004	0.156	0.876	0.001 (−0.007, 0.008)	0.004	0.330	0.742	0.001 (−0.007, 0.009)
3-5MVM types	0.008	−2.344	0.019	−0.020 (−0.036, −0.003)	0.009	−2.185	0.029	−0.019 (−0.036, −0.002)

^a^ Linear regression analysis adjusted for Maternal age, BMI, before delivery, hypertensive disorders of pregnancy, Hypothyroidism, gestational diabetes mellitus, and Intrahepatic cholestasis of pregnancy. MVM, maternal vascular malperfusion. crSO_2_, cerebral regional oxygen saturation; cFTOE, cerebral fractional tissue oxygen extraction.

#### 3.4.2 cFTOE during the first postnatal week

Univariate linear regression analysis showed that compared with no MVM, 3-5 types of MVM group were associated with lower cFTOE within the first week after birth (β = −0.020, 95% CI: −0.036 to −0.003, P = 0.019), while 1-2 types of MVM were not significant. After adjusting for confounding factors such as gestational diabetes mellitus, gestational hypertension, and hypothyroidism, the presence of placental findings of 3-5 types of MVM was associated with lower cFTOE within the first week of life compared with no MVM (β = −0.019, 95% CI: −0.036 to −0.002, P = 0.029) (Table 6).

## 4 Discussion

MVM can disrupt fetal oxygen supply and contribute to severe neurological complications, such as reduced fetal brain volume (Oh et al., 2021). By monitoring cerebral tissue oxygenation status in neonates during the neonatal period, this study systematically investigated the dynamic association between the severity of maternal placental MVM pathology (quantified by the number of MVM lesion types) and neonatal cerebral oxygen metabolism. Key findings revealed that neonates with 3–5 types of MVM lesions in the maternal placenta exhibited significantly higher crSO_2_ and lower cFTOE within the first postnatal week, compared to those with fewer or no MVM lesions. These results suggest that severe MVM exerts a distinct impact on cerebral oxygen saturation in early neonates.

In the present study, neonates with 3-5 types of MVM had a slightly lower median gestational age (33.36 weeks) compared to those in the no MVM group (34 weeks) and the 1-2 types of MVM group (34 weeks). Placental dysfunction is strongly associated with preterm birth (Brink et al., 2022; Visser et al., 2021), a finding consistent with a prior study of 728 patients with gestational hypertension that reported a decreasing MVM incidence with advancing gestational age (95.4% at <34 weeks, 69.8% at 34–36 weeks, and 50% at ≥37 weeks) (Freedman et al., 2023). Collectively, these observations support the conclusion that severe MVM constitutes a critical pathological substrate for preterm birth.

In addition, neonates in the 3-5 types of MVM group had a lower median birth weight (1,695 g) compared to those in the no MVM group (1840 g) and the 1-2 types of MVM group (1880 g), suggesting a strong association between MVM and fetal intrauterine growth restriction (Zur et al., 2024). Notably, while no statistically significant difference was observed in the incidence of small for gestational age across the three groups (P = 0.123)—a finding potentially attributed to limited sample size—the numerical trend in birth weight still supports the presence of an underlying association between MVM severity and fetal growth impairment.

With respect to maternal complications, the incidence of HDP in the 3-5 types of MVM subtypes group was 71.43%, which was significantly higher than that in the 1-2 types of MVM subtypes group (44.2%) and the no MVM group (40.37%). This finding aligns with current pathophysiological understanding: the pathological basis of HDP lies in insufficient spiral artery remodeling caused by impaired trophoblast invasion, whose placental manifestation is MVM, with the incidence of MVM positively correlated with the severity of HDP (Weiner et al., 2018). Notably, a study by Richter et al. reported an association between preeclampsia and lower cFTOE in early neonates, which is consistent with the results of the present study (Richter et al., 2020).

In the present study, neonates with 3-5 types of MVM exhibited a cerebral oxygenation profile characterized by elevated crSO_2_ and reduced cFTOE during the early neonatal period. This seemingly paradoxical observation—chronic intrauterine hypoxia associated with postnatal increases in cerebral oxygen saturation—could potentially be interpreted as reflecting persistence of fetal cerebral blood flow compensatory mechanisms into the neonatal period. To counteract chronic intrauterine hypoxia, the fetus activates a “brain-sparing effect” that prioritizes cerebral perfusion over other organ systems. Such hemodynamic adaptations may persist postnatally for a transient period. For instance, prior research has demonstrated that preterm infants maintain higher cerebral blood flow even at corrected gestational age compared to term infants (Bouyssi-K et al., 2018). In the current study, the 3-5 types of MVM group had a slightly lower median gestational age than other groups, and their elevated cerebral oxygen saturation may reflect a more robust cerebral blood flow regulatory state linked to their younger gestational age. In essence, the presence of 3-5 types of MVM may induce a fetal cerebral adaptive pattern of “hyperperfusion with reduced oxygen extraction,” which persists transiently in the early postnatal period.

In the present study, crSO_2_ levels in all neonates exhibited a downward trend with increasing postnatal age, while cFTOE levels gradually increased—consistent with findings reported by Mohamed et al. (2021). Notably, neonates with placental findings of MVM (particularly those with 3-5 types) had higher crSO_2_ than those in the no MVM group during the first two postnatal weeks, but lower crSO_2_ in later weeks; the magnitude of this crSO_2_ shift was positively correlated with MVM severity. This observation suggests that the early cerebral hyperperfusion induced by MVM may resolve as vascular regulatory capacity normalizes, potentially unmasking latent effects on cerebral oxygen metabolism over time. Differences in cFTOE across the three groups reached statistical significance only during the first postnatal week. Longitudinal analyses of the data revealed that neither the between-group main effect nor the time × group interaction effect reached statistical significance. These findings collectively indicate that intergroup differences in cerebral oxygenation were primarily restricted to the early neonatal period, rather than persisting longitudinally.

The present study advances understanding by elucidating the cumulative effect of placental MVM lesions. Herein, we quantified the “cumulative burden of placental dysfunction” using the number of distinct MVM lesion subtypes present in the mother, and for the first time, correlated this metric with neonatal cerebral oxygenation parameters (crSO_2_, cFTOE). Our findings indicate that when MVM lesions accumulate to three or more subtypes, they exert a significant impact on the cerebral tissue oxygenation status of offspring. This work suggests that a simplified quantitative assessment of placental pathology reports—specifically, enumerating the number of MVM lesion subtypes—may provide useful postnatal contextual information for interpreting early cerebral oxygenation patterns in neonates. Recognition of a higher cumulative MVM burden on placental examination may help identify neonates who could benefit from closer cerebral oxygenation monitoring during the first postnatal week, a critical period of physiological transition.

The present study, while offering valuable insights, is not without its limitations. One of the primary constraints lies in the relatively small size of the sample. When we take a closer look at the data, the issue becomes even more pronounced, particularly in the 3 - 5 types of MVM group. The number of cases is notably scarce. In statistical analysis, a small sample size may lead to a lack of statistical power. With a limited number of cases in the 3 - 5 types of MVM group, there is a heightened risk of type II errors. Although we observed statistically significant associations in the multiple regression model, we must state that this finding is mainly based on a small sample subgroup. Therefore, this association conclusion should be regarded as preliminary, highlighting the possible important link between the cumulative load of MVM and early cerebral oxygenation patterns, but the effect strength and generalizability still need to be confirmed through prospective studies with expanded samples, especially by increasing the number of severe MVM cases.

Another limitation of this study is that other placental pathological categories, including fetal vascular malperfusion (FVM) and maternal or fetal inflammatory responses (MIR/FIR), were not included in adjusted or sensitivity analyses. We explored the feasibility of incorporating FVM and inflammatory lesions into sensitivity analyses; however, due to very low event numbers and non-standardized reporting, such analyses were considered statistically unreliable and were therefore not presented. In addition, data on these lesions were derived from routine pathology reports, were not systematically graded or quantified, and showed low prevalence with no significant differences across MVM burden groups (Supplementary Table S1). Under these conditions, further adjustment was unlikely to meaningfully alter effect estimates and could have compromised model stability, particularly given the small sample size in the highest MVM burden group. Nevertheless, residual confounding by other placental pathologies cannot be completely excluded, underscoring the need for future prospective studies with standardized placental phenotyping.

It is important to note this is a retrospective observational study. A potential selection bias exists in this study. The neonates born to the included mothers who underwent placental pathological testing limit the generalizability of the results to the entire neonatal population—particularly to healthy full-term infants without overt clinical risk factors. This skews our study cohort toward mother-infant dyads with underlying risks or comorbidities. Retrospective observational studies rely on existing data. While they can provide a wealth of information based on real-world scenarios, they also come with inherent limitations. The current monitoring interval time for brain tissue oxygen saturation in this study may not be optimal. By having a relatively long monitoring interval, we might be missing out on more subtle changes in brain tissue oxygen saturation during the neonatal period.

Furthermore, preterm birth is widely recognized as one of the primary clinical outcomes of severe MVM, with a well-established bidirectional association between the two. As such, gestational age was not forcedly adjusted as a confounder in our primary analytical model—this decision was made to avoid over-adjustment bias and prevent masking the changes in cerebral tissue oxygen saturation (ctSO_2_) directly driven by MVM itself. However, we acknowledge that this approach introduces a critical analytical challenge: it hinders the complete disentanglement of MVM’s direct effects on ctSO_2_ from its indirect effects mediated through preterm birth. To address this limitation, future prospective studies should employ more refined study designs (e.g., gestational age matching or stratification by preterm status) in larger cohorts to further dissect the causal pathways underlying this relationship.

## 5 Conclusion

An association was observed between the presence of 3-5 types of maternal vascular malperfusion (MVM) and altered cerebral oxygenation parameters during the early postnatal period. These findings indicate that the cumulative burden of MVM lesions identified on postpartum placental examination may provide useful contextual information for interpreting early neonatal cerebral oxygenation patterns and highlight the need for closer physiological observation during the first postnatal week.

## Data Availability

The datasets presented in this article are not readily available because The datasets used and analysed during the current study available from the corresponding author on reasonable request. Requests to access the datasets should be directed to tao_xiong@126.com.
