# Supplementary material for: Cumulative burden of maternal vascular malperfusion and its association with early cerebral oxygenation in neonates
Source: Front Cell Dev Biol. 2026 Feb 11;14:1756278. doi: 10.3389/fcell.2026.1756278 (PMC12932476; doi:10.3389/fcell.2026.1756278)
Supplement: Supplementary file 1 [file Table1.docx]

***Supplementary Table 1***

Prevalence of selected fetal vascular malperfusion (FVM) and maternal and fetal inflammatory placental lesions (MIR/FIR) across different maternal vascular malperfusion (MVM) burden groups.

| Variables | No MVM (n=161) | 1–2 MVM types (n=319) | 3–5 MVM types (n=28) | *P* |
| --- | --- | --- | --- | --- |
| MIR |  |  |  |  |
| Acute subchorionitis, n (%) | 5 (3.11) | 10 (3.13) | 0 (0.00) | 1.000 |
| Severe chorioamnionitis, n (%) | 15 (9.32) | 19 (5.96) | 1 (3.57) | 0.302 |
| FIR |  |  |  |  |
| Necrotizing funisitis, n (%) | 1 (0.62) | 1 (0.31) | 0 (0.00) | 1.000 |
| Umbilical arteries, n (%) | 4 (2.48) | 4 (1.25) | 0 (0.00) | 0.652 |
| Umbilical phlebitis, n (%) | 7 (4.35) | 5 (1.57) | 0 (0.00) | 0.179 |
| Chorionic vasculitis, n (%) | 3 (1.86) | 4 (1.25) | 0 (0.00) | 0.793 |
| FVM |  |  |  |  |
| Thrombosis, n (%) | 1 (0.62) | 6 (1.88) | 0 (0.00) | 0.619 |

Data on FVM and inflammatory lesions were retrospectively extracted from available routine placental pathology reports. These lesions were not systematically staged, graded, or quantified according to standardized criteria, and their overall prevalence was low with no significant differences observed across MVM burden groups. We explored the feasibility of incorporating these lesions into adjusted or sensitivity analyses; however, due to very low event numbers and non-standardized reporting, such analyses were deemed statistically unreliable and are therefore not presented. Accordingly, the data in this table are provided for descriptive purposes only.
